# Rare variants in embryonic development and cell signalling genes in syndromic and non-syndromic orofacial clefts: evidence from a Colombian Caribbean cohort

**DOI:** 10.1038/s10038-026-01466-x

**Published:** 2026-03-30

**Authors:** Alejandro Silva, Carolina Jaramillo Oquendo, Jaime E. Bernal, Julio Cesar Martinez, Andrew Collins, Ignacio Briceño, Escilda Benavides, Zulieth López Arrieta, Sarah Ennis

**Affiliations:** 1 Human Genetics and Genomic Medicine, University of Southampton, Southampton, UK; 2 Grupo de Genética Humana, Facultad de Medicina—Universidad de La Sabana, Chía, Colombia; 3 Grupo Genoma—Facultad de Medicina, Universidad del Sinú, Cartagena, Colombia

**Keywords:** Risk factors, Disease genetics, Genetics research, Disease genetics

## Abstract

Orofacial clefts (OFCs) are common craniofacial malformations broadly classified as syndromic or non-syndromic. While syndromic OFCs are often caused by rare, high-impact variants, non-syndromic OFCs are typically associated with multiple low-impact common variants. However, growing evidence suggests that rare variants may also contribute to non-syndromic OFCs. To explore this, we performed exome sequencing in 45 individuals from 20 Colombian families, predominantly from the Caribbean region, a genetically distinct and underrepresented population. Our goal was to identify rare variants potentially contributing to both syndromic and non-syndromic OFCs. We identified 15 rare protein-altering variants in 11 families that showed strong phenotype-genotype concordance. Four probands carried a previously reported common *ACSS2* variant (c.1487 T > C), with two probands also harbouring variants in Pleckstrin Homology Domain Containing *(PLEKH*) genes. Five variants were previously reported in ClinVar (two with conflicting interpretations, one pathogenic, and two of uncertain significance), while ten were novel. Variants were found in known OFC-associated genes (*MID1, FLNA, FGF10*) and emerging candidates (*ZFHX4, PLEKHA5, PLEKHA7*). These findings provide further evidence that rare variants in developmental and signalling pathways contribute to both syndromic and non-syndromic OFCs, reinforcing previous studies and expanding the catalogue of candidate genes in underrepresented populations.

## Introduction

Orofacial clefts (OFCs) are common congenital anomalies generally divided into two distinct types: cleft lip with or without cleft palate (CL/P) and cleft palate only (CPO). These entities differ in embryological origin and genetic risk factors. While CL/P and CPO represent the most prevalent forms, other less common types—such as submucous cleft palate, median cleft lip, and atypical facial clefts—also exist and may present with distinct clinical and genetic features. Also, OFCs may occur as non-syndromic, appearing in isolation, or as syndromic, accompanied by other anomalies such as heart defects, hearing loss, or neurodevelopmental disorders [1]. Non-syndromic OFCs (nsOFCs) account for nearly 70% of all OFCs and are considered complex multifactorial disorders whose frequencies vary by ethnic origin and socio-economic status [2].

Syndromic OFCs often follow a classic Mendelian inheritance pattern and are caused by rare genetic changes with significant effects, such as single nucleotide variants (SNVs) in critical genes (e.g., *IRF6*, *NECTIN1*, *CDH1*, *MSX1*), copy number variants (CNVs), or chromosomal abnormalities [1]. On the other hand, non-syndromic OFCs are typically associated with multiple low-impact common variants, although rare variants with variable penetrance and expressivity may also contribute [3, 4]. Furthermore, genes initially associated with syndromic orofacial clefts—such as *IRF6*, *GRHL3*, *TFAP2A*, and *CDH1*—have also been implicated in non-syndromic presentations [5, 6]. Additionally, genome-wide association studies suggest that common variants contribute more substantially to non-syndromic cleft lip with or without cleft palate (nsCL/P) than to non-syndromic cleft palate only (nsCPO). While over 40 genome-wide significant loci have been identified for nsCL/P, fewer than 10 have been reported for nsCPO, despite similar cohort sizes—highlighting distinct genetic architectures between the two phenotypes [7].

Most large-scale studies on genotype–phenotype associations have focused on European populations, identifying key loci and genes such as 8q24, *IRF6*, *PAX7*, *VAX1*, and *MAFB*, accounting for up to 55% of the attributable risk in these cohorts [1]. However, genome-wide association studies (GWAS) are limited in their ability to pinpoint causal variants and fully explain OFC heritability, prompting a shift toward exome sequencing (ES) to identify rare variants.

Studies in African, South Asian, East Asian, and Middle Eastern populations have identified both common and rare variants in genes such as *ARHGAP29, ABCA4, MSX1, CHD7, FGFR2*, and *NRP1*, highlighting the genetic heterogeneity of OFCs [8–10]. Among Latino populations, particularly in Brazil and Colombia, 8q24, *IRF6*, and *MSX1* have been associated with increased OFC risk [11–13]. Recent genetic studies in Colombian populations have also revealed a complex and population-specific architecture with variants in genes such as *IRF6* [12], *MSX1* [13], and *CRISPLD2* [14] and founder mutations like those in *PVRL1* observed in Antioquian families [15]. Also, exome sequencing and genome-wide association studies have identified novel candidate loci and rare variants unique to Colombian cohorts [16, 17]. Additional genes such as *ARHGAP29* [18], *MAFB* [19], *PDGF-C* [20], and *ACSS2* [21] have shown relevance in Colombian individuals with OFC, further supporting the role of both common and rare variants.

Even though OFC studies conducted on the Colombian population have advanced the understanding of the genetic causes of this condition, the population of the Caribbean coast remains poorly represented. This region has a unique genetic background shaped by African and Arab ancestry, unlike the predominantly European Ancestry of the Andean region [22]. Moreover, access to advanced genetic testing remains limited in Colombia, as in many low- and middle-income countries, with diagnosis often relying on prenatal ultrasound and basic cytogenetic methods [23].

To address this gap, we performed exome sequencing in 45 individuals from 20 Colombian families primarily from the Caribbean departments of Bolívar and Guajira, and the Andean departments of Cundinamarca and Boyacá. Our aim was to identify rare variants contributing to both syndromic and non-syndromic OFCs in this genetically distinct and understudied population.

## Materials and methods

### Cohort

Participants were recruited between 2019 and 2022 in three different settings: (1) routine genetics clinic at Operation Smile Foundation in Bogotá; (2) a health campaign in the city of Riohacha, Guajira, in coordination with Operation Smile Foundation and; (3) a recruitment campaign in the municipality of Mompox, Bolívar in coordination with municipal health authorities. The study included 45 participants from five departments in Colombia. From the Andean region, 11 participants were from Cundinamarca and 4 from Boyacá. The remaining 30 participants (67%) came from the Caribbean region, including Bolívar (*n* = 24), La Guajira (*n* = 5), and Cesar (*n* = 1).

Probands were recruited if they had any type of OFC, as determined by a clinical geneticist. The cohort included individuals with both syndromic and non-syndromic cleft lip with or without cleft palate (CL/P), as well as two syndromic individuals with cleft palate only (CPO). Cleft phenotypes were classified as bilateral or unilateral cleft lip and palate (CLP), or as isolated cleft lip (iCL). A comprehensive clinical genetics medical history was documented for each family, recording sex, family background, prenatal information, and comorbidities. Additionally, each subject underwent a thorough physical examination to confirm their diagnosis, with findings recorded in their medical records. This included details on the type and laterality of cleft lip and/or palate, other congenital malformations, and any additional clinical observations. Patients provided laboratory results, imaging tests, and other medical records as requested by specialists from the Operation Smile Foundation team, such as paediatricians, cardiologists, plastic surgeons, and speech therapists.

Ethical approval was obtained from the Institutional Ethics Committee of Clínica de la Sabana (IEC Session #82, 13/11/2019) based in Chía, Cundinamarca. All participants provided signed informed consent, either by themselves or their legal guardians (for those who were under 18 years old). Once informed consent was signed and blood sample taken, each participant received a study-specific code with which all of their personal and medical information was anonymised and stored in a password-protected electronic folder.

### DNA extraction and exome sequencing

Blood samples were taken during the recruitment visit by venepuncture with a BD Vacutainer system, using whole blood/plasma evacuated tubes (BD Vacutainer® Plus K_2_EDTA Tubes) and sent to GenCell Pharma (Bogotá, Colombia) for genomic DNA extraction, NGS library preparation and sequencing. Extraction was performed using an automated magnetic bead purification method and an EXM3000 Nucleic Acid Isolation System (Zybio^®^). Library preparation was done with MGIEasy Exome Capture V5 Probe Set, which is designed to enrich exons in human protein-encoding genes, short flanking intronic regions, miRNA genes and mtDNA. Next generation paired-end sequencing was carried out using DNBSEQ Technology in a DNBSEQ-G400 sequencer (MGI^®^), which is based on the generation of DNA nanoballs (DNBs) for each DNA fragment created in the library and binding of each DNB on a patterned silicon chip. (https://en.mgi-tech.com/products/resources).

### Bioinformatic analyses

Samples were processed according to GATK’s best practice recommendations [24]. Raw sequencing FASTQ files were processed through FastQC v0.11.5 (https://www.bioinformatics.babraham.ac.uk/projects/fastqc/) for quality control. Alignment was performed against the human reference genome (GRCh38/hg38 Dec. 2013 assembly) using BWA-mem version 0.7.15 [25] and sorted and indexed using Samtools v1.2.3 [26]. Duplicate reads were marked using Picard Tools v1.97 (http://broadinstitute.github.io/picard). Variants were called using GATK HaplotypeCaller in GVCF mode to produce a gVCF file for each sample. GATK GenomicsDBImport was used to merge GVCFs from all 45 samples for input into GATK GenotypeGVCFs producing joint-called SNP and indels calls ready for filtering. Variant quality score recalibration (VQSR) was carried out using GATK VariantRecalibrator and ApplyVQSR. GATK VariantAnnotator was used to annotate joint VCF with pedigree information to extract high confidence de novo calls in families with sufficient information. Finally, variants were annotated using ensemble-vep v111 [27] adding a gene-based annotation to identify functional effects, frequency of the variant in gnomAD v4.1.0 [28], and predictive scores including SpliceAI [29], CADD [30] AlphaMissense [31] and Revel [32]. The versions of the pre-computed scores (e.g., SpliceAI, CADD) correspond to those supported with emsembl-vep v111 at the time of annotation.

### Sample QC and ancestry analyses

Somalier [33] was used to confirm expected sample relatedness across families, perform ancestry estimation using principal component analysis, and check concordance between genetically determined and reported sex. Relatedness is calculated by using identity-by-state (IBS) metrics derived from 17,766 sites within the coding sequence. Using these sites, Somalier calculates total counts with zero shared alleles (IBS0), total counts where both samples have the same genotype, shared heterozygous and homozygous alternate sites, and per-sample heterozygosity. The relatedness between sample i and j is then calculated as *(shared-hets(i,j)-2 x ibs0(i,j))/min(hets(i),hets(j))*. The 17,766 sites also include variants on the X and Y chromosomes which are used to estimate sample’s sex from genotype but are not used for the relatedness calculations. Ancestry estimation was performed with Somalier using 2504 labelled samples from the 1000 Genomes Project to construct the principal component analysis (Supplementary Fig. S1).

### Variant prioritisation

To prioritise variants, we took two complementary approaches. First, we applied a genotype-to-phenotype strategy, where the annotated joint VCF was run through an in-house script to exclude:

- Variants with a minor allele frequency >0.01 in gnomAD v4.1.0 (any population),
- Variants in non-coding regions,
- Synonymous variants not predicted to affect splicing (SpliceAI score < 0.2),
- Variants with a CADD score <20.

Both SpliceAI and CADD score cutoffs are the recommended thresholds by the authors of the tools [29, 30].

To focus on genes with known disease associations, the Clefting v6.0 and Paediatric Disorders v52.5 (R27) gene panels from the UK Genomic Medicine Service (GMS) PanelApp resource were applied [34].

**Fig. 1:**
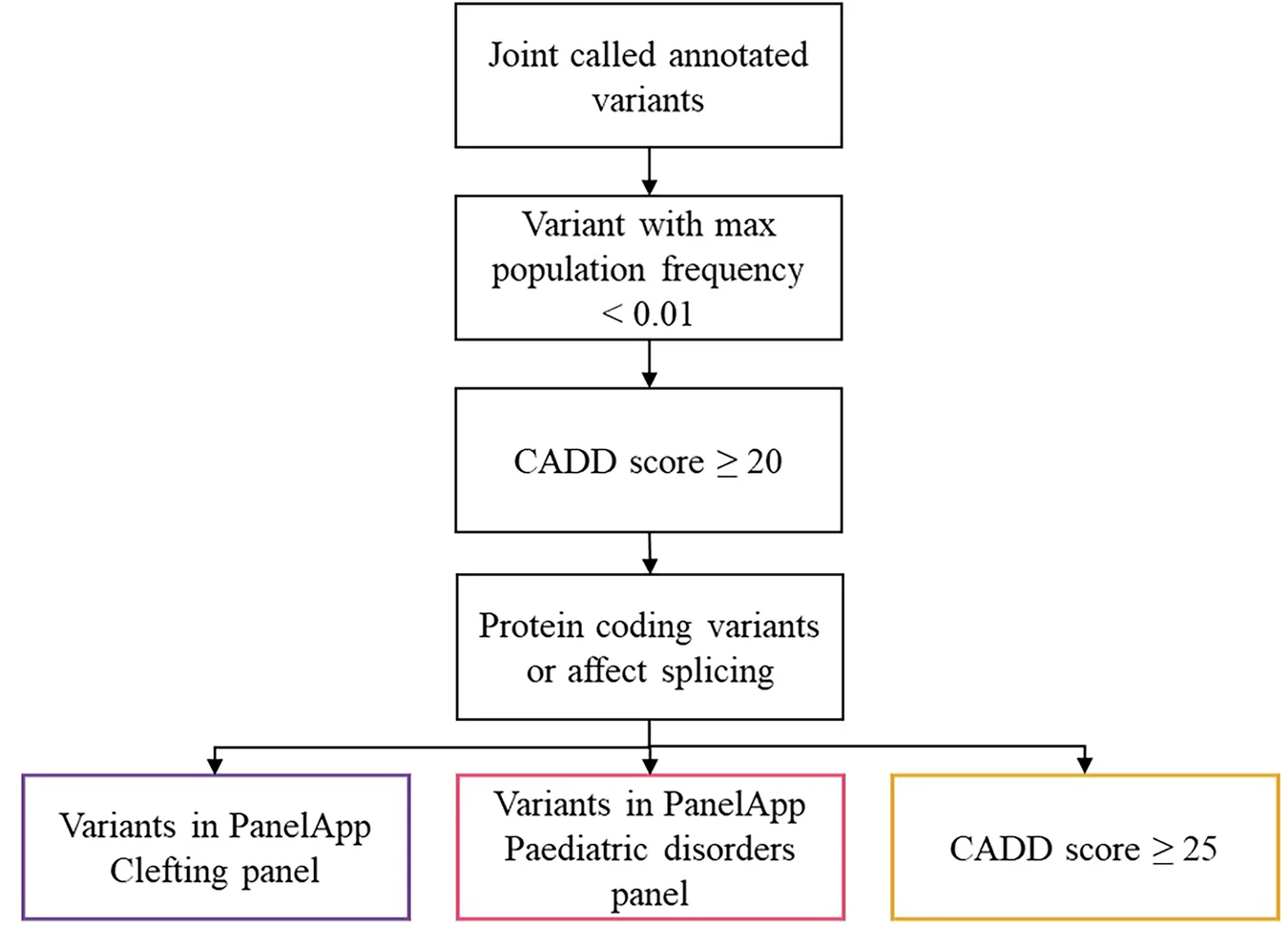
Filtering strategies using a genotype to phenotype approach. The joint annotated VCF was filtered based on minor allele frequency and CADD score. Subsequently, variants were selected if they were in genes found in the Clefting and Paediatric Disorders (R27) panels. Additionally, any variant with a CADD score ≥ 25 was also selected

Recognising that our cohort come from an underrepresented population in genomic databases, we performed and additional inclusive filtering step to identify potential novel genes contributing to OFC in this population. This included:

- Variants with a minor allele frequency (MAF) ≤ 0.01,
- Variants located in the coding region (including synonymous variants predicted to affect splicing (SpliceAI score ≥0.2)),
- Variants with a CADD PHRED score ≥25 (Fig. 1).

Our second approach involved the use of the Franklin variant interpretation platform (https://franklin.genoox.com/clinical-db/home) to broaden our analysis and ensure a more inclusive methodology. This approach allowed us to identify potential variants beyond those included in currently available gene panels, which are still evolving and may not yet capture all relevant disease-related genes. Individual and family VCF files were loaded to Franklin to perform individual or family-based analysis. Two different filtering strategies were performed across all probands: (1) phenotype-based gene lists where proband HPO terms were used to generate a list of genes associated with the phenotype and; (2) pathogenic/likely pathogenic variants reported in ClinVar. A description of all parameters used in Franklin are found in Table 1.

**Table 1:** Filtering strategies used in Franklin and associated parameters

	Strategy 1		Strategy 2
	P/LP/VUS variants associated with phenotypic terms		ClinVar Pathogenic
	Quartets/Trios	Individuals/Duos	
Phenotype-based gene list	Yes	Yes	NA
Inheritance	AR, AD, De novo, X linked, Y linked^1^	NA	NA
Franklin Classification	P, LP, VUS	P, LP, VUS	NA
Region	Exonic, Canonical Splice Site, ± 3–10	Exonic, Canonical Splice Site, ± 3–10	NA
Effect	Synonymous, Missense, Stop gain/loss, Start gain/loss, frameshift, Inframe indel	Synonymous, Missense, Stop gain/loss, Start gain/loss, frameshift, Inframe indel	NA
Proband Zygosity	Homozygote, Heterozygote	Homozygote, Heterozygote	NA
Family Zygosity	Homozygous ref in sibling^2^	NA	NA
Variant allele frequency	0.25–1.00	0.25–1.00	0.15–1.00
Minor allele frequency	gnomAD ≤0.05	gnomAD ≤0.05	gnomAD ≤ 0.05
Confidence^3^	Low, Medium, High	Low, Medium, High	Low, Medium, High
ClinVar	Exclude B/LB	Exclude B/LB	Only P/LP

Two strategies were used to filter variants: 1. All pathogenic/likely pathogenic variants and variants of uncertain significance (VUS) in a gene list created based on HPO terms describing the phenotype of each proband and (2) all pathogenic and likely pathogenic variants reported in ClinVar

1. Assuming complete penetrance, 2. For quartets to identify the proband’s exclusive variants, 3. Franklin’s confidence criteria.

*P* pathogenic, *LP* likely pathogenic, *VUS* variant of uncertain significance, *LB* likely benign, *B* Benign, *NA* not applicable, *AR* autosomal recessive, *AD* autosomal dominant, *ref* reference

Finally, variants shortlisted by either approach were manually reviewed in IGV and prioritised considering cohort frequency, variant phasing where possible, gene-to-phenotype concordance, ClinVar classification, and variant curation based on ACMG/AMP guidelines. Where trio/quartet data allowed, segregation analysis was performed per family and de novo variants where investigated.

## Results

The final cohort comprised 45 participants across 20 families in Bolivar, Guajira and Bogotá, 22 of which were affected individuals with OFC phenotypes. The cohort included three quartets (mother, father, proband and sibling), five trios, six duos, and six individual probands (Fig. S2). Demographic data is summarised in Table 2. Six probands had family history of OFC (30% of the cohort), while the remaining were sporadic occurrences.

**Table 2:** Demographic data for the 20 recruited families

Metric	All individuals	Affected	Unaffected
Number of samples	45	22	23
Males	17	9	8
Females	28	13	15
Individuals with orofacial surgery	21 (46%)	21 (95%)	0 (0%)
**Type of OFC**			
nsCL/P		12	
sCL/P		8	
sCPO		2	

*CL/P* cleft lip with or without cleft palate, *nsCL/P* non-syndromic CL/P, *sCL/P* syndromic CL/P, *sCPO* syndromic cleft palate only

Of the 22 probands, 12 presented as non-syndromic and 10 as syndromic OFC (Supplementary Table S1). Among syndromic individuals, additional phenotypes included cardiac (30%), neurodevelopmental (50%), growth and skeletal (70%), and abdominal anomalies (20%). Genitourinary, ocular, respiratory, immune system and facial anomalies were observed in one proband each. Supplementary Table S2 summarizes the clinical information of the individuals in our cohort.

Samples had an average of 71 million uniquely mapped reads, with a mean target coverage of 80x (Supplementary Fig. S1A, B). The relatedness coefficients generated by Somalier were consistent with reported pedigrees, except for one discordant pair (C17-C18). Initially labelled as parent-child, follow-up revealed they were not biologically related. Genetic data helped resolve potential mislabelling. Participant C18 was confirmed to be the father of proband C16, but not of C17. Instead, C17 was identified as the half sibling of proband C16. These labelling discrepancies did not affect downstream analyses. Reported sex and genotype-inferred sex were concordant for all samples (Supplementary Fig. S1C, D). Our samples clustered within the Admixed American (AMR) group when analysed according to the five major human genetic superpopulations (AFR, AMR, EAS, EUR, and SAS). The AMR labels assigned by Somalier are consistent with the known genetic diversity of admixed American populations.

### Exome sequencing identifies candidate variants in 11 OFC probands

**Fig. 2:**
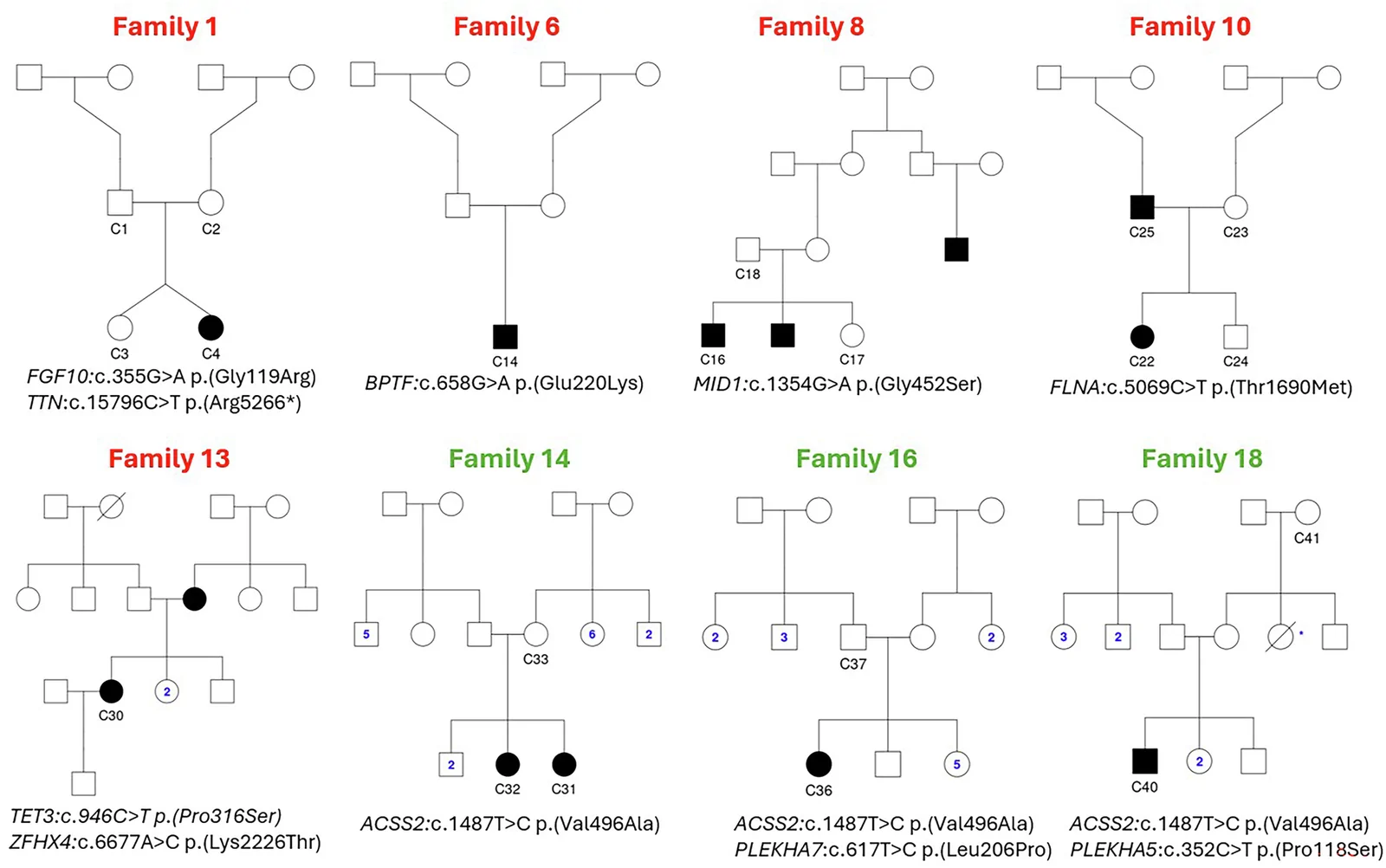
Pedigrees of selected families. ID codes for research subjects appear under each square/circle, individuals whose DNA was collected and analysed. Symbols filled in black represent affected individuals. Numbers inside of circles and squares represent the number of brothers and sisters, respectively. Labels in red indicate the family includes individual(s) with sOFC, while labels in green indicate families with individual(s) with nsOFC. Under each pedigree, the HGVS nomenclature of the variants found in the respective affected individuals of each family is included. For family 14, only the *ACSS2*:c.1487 T > C variant was found in the three individuals analysed: C31, C32 and C33

After filtering (genotype-to-phenotype approach + Franklin) and manual curation, we identified 15 variants (13 missense, 1 stop gain, and 1 indel) across 11 families, each with a potential link to the proband’s phenotype (Table 3). ES helped identify candidate variants in 40% and 58% of the sOFC and nsOFC individuals, respectively. Notably, the variant *ACSS2*:c.1487 T > C, classified as VUS (PS4, PP1, PP3, BS2), was identified in four probands across families 14, 16 and 18 but also in the unaffected sister from Family 8. In Family 14, this variant was found in both affected sisters with non-syndromic OFC and was absent from their unaffected mother. Likewise, in Families 16 and 18, the variant was absent in the proband’s unaffected parents. Pedigrees of these families and for the families mentioned below can be seen in Fig. 2.

**Table 3:** Selected variants identified using both filtering approaches (gene-to-phenotype and Franklin) after manual curation

OFC type	Family ID	Sample ID	Variant	Symbol	Zygosity	Variant inheritance	Filtering strategy	HGMD Class	Alpha missense	REVEL	CADD PHRED
sOFC	1	C4	NM_001267550.2:c.15796 C > T p.(Arg5266*)	*TTN*	het	de novo	R27; CADD 25	P	-	-	38
sOFC	1	C4	NM_004465.2:c.355 G > A p.(Gly119Arg)	*FGF10*	het	de novo	R27; CADD 25; Franklin 2	LP	0,9975	0,867	33
sOFC	4	C12	NM_001170535.3:c.968 G > T p.(Ser323Ile)	*ATAD3A*	het	unknown	R27	VUS	0.119*	0,382	20,5
sOFC	4	C12	NM_006734.4:c.5312 A > G p.(Glu1771Gly)	*HIVEP2*	het	unknown	R27	VUS	0,6935	0,233	26,3
sOFC	6	C14	NM_182641.4:c.658 G > A p.(Glu220Lys)	*BPTF*	het	unknown	R27; CADD 25	VUS	0,982	0,446	30
sOFC	8	C16	NM_000381.4:c.1354 G > A p.(Gly452Ser)	*MID1*	hemi	XLR	Clefting; R27	VUS	0,9709	0,442	24,8
nsOFC	10	C22	NM_001110556.2:c.5069 C > T p.(Thr1690Met)	*FLNA*	het	XLD	Clefting; R27; Franklin 1	VUS	0,1451	0,807	23,4
nsOFC	12	C29	NM_002633.3:c.734 C > T p.(Ala245Val)	*PGM1*	hom	unknown	Clefting; CADD 25; Franklin 1	VUS	0,1378	0,228	26,6
nsOFC	13	C30	NM_001287491.2:c.946 C > T p.(Pro316Ser)	*TET3*	het	unknown	R27	VUS	0,085	0,851	20,7
nsOFC	13	C30	NM_024721.5:c.6677 A > C p.(Lys2226Thr)	*ZFHX4*	het	unknown	R27	VUS	-	-	24,4
nsOFC	15	C34	NM_000138.5:c.1125 G > T p.(Glu375Asp)	*FBN1*	het	unknown	R27; Franklin 1	VUS	0,4406	0,357	20,4
nsOFC	15	C34	NM_138927.4:c.78-4_78del p.(?)	*SON*	het	unknown	Clefting; Franklin 1	LP	-	-	-
nsOFC	16	C36	NM_001329630.2:c.617 T > C p.(Leu206Pro)	*PLEKHA7*	het	unknown	Clefting, R27; CADD 25	VUS	0,9855	0,681	25,2
nsOFC	18	C40	NM_001256470.2:c.352 C > T p.(Pro118Ser)	*PLEKHA5*	het	unknown	Clefting; R27	VUS	0,1751	0,115	22,2
nsOFC	14, 16, 18	C31, C32, C36, C40	NM_018677.4:c.1487 T > C p.(Val496Ala)	*ACSS2*	het	AD	Franklin 2	VUS	0,4498	0,566	31

This list includes variants associated with OFC and one incidental finding (in TTN)

*: Alpha Missense score NOT from MANE select

*OFC* orofacial cleft, *sOFC* syndromic OFC, *nsOFC* non-syndromic OFC, *AD* autosomal dominant, *XLR* x-linked recessive, *XLD* x-linked dominant, *P* pathogenic, *LP* likely pathogenic, *VUS* variant of uncertain significance, *het* heterozygous, *hemi* hemizygous, *hom* homozygous

According to ACMG criteria, of the 15 unique variants, one was classified as pathogenic, two as likely pathogenic and 12 as variants of uncertain significance (VUSs) (Supplementary Table S3). Ten variants had previous entries in dbSNP, five of which were also listed in ClinVar with disease associations (Supplementary Table S4). Clinvar entries included two conflicting interpretations of pathogenicity, one pathogenic and two VUS. The remaining 10 variants are novel findings and have not been previously linked to any condition. All variants identified before manual curation are found in the Supplementary Tables S5.

### Variants in genes with established links to OFC

We identified variants in genes with an established link to OFCs or strong candidate genes with supporting functional evidence. The most notable variants were found in early development genes *MID1* [35, 36], *FLNA* [37] and *FGF10* [38, 39].

A missense VUS in *MID1*, NM_000381.4:c.1354 G > A p.(Gly452Ser), was identified in proband C16 [family 8], who presented with syndromic OFC, delayed speech and language development, mild facial dysmorphism and short stature. In proband C22 [family 10], a female individual with a syndromic phenotype involving cardiac, ocular and neurodevelopmental features, we identified a heterozygous missense variant in *FLNA*, NM_001110556.2:c.5069 C > T p.(Thr1690Met), inherited from the affected father. The variant was absent in the unaffected mother and sibling and was present in a hemizygous state in the affected father (C25). Proband C4 [Family 1] harboured two de novo mutations, a missense likely pathogenic variant in *FGF10*, NM_004465.2:c.355 G > A p.(Gly119Arg), and a pathogenic variant in *TTN*, NM_001267550.2:c.15796 C > T p.(Arg5266*).

In proband C14 [family 6], we detected a missense VUS in *BPTF*, NM_182641.4:c.658 G > A p.(Glu220Lys), while in proband C29 [family 12], a non-syndromic individual, we identified a homozygous VUS in *PGM1*, NM_002633.3:c.734 C > T p.(Ala245Val).

### Variants in emerging or novel candidate OFC genes

This work also identified variants within emerging candidate genes linked to OFC including *ZFHX4* [40], *PLEKHA5* and *PLEKHA7* [4].

In proband C30 [Family 13], an individual with syndromic features including complete unilateral cleft lip, clinodactyly and dental crowding, we identified a heterozygous missense variant within the *ZFHX4* gene, NM_024721.5:c.6677 A > C p.(Lys2226Thr). Notably, this proband also harboured a variant within *ZFHX3*, NM_006885.4:c.8446 C > T p.(Pro2816Ser), a paralog of *ZFHX4*. Both genes encode transcription factors that play roles in regulating gene expression during development. In gnomAD v4.1.0, NM_006885.4:c.8446 C > T is observed in a single individual of East Asian ancestry. Based on ACMG classification criteria, we classified this variant as likely benign and therefore excluded it from the final list of prioritised variants.

In proband C36 [Family 16], a non-syndromic individual, we identified two variants of interest: the recurrent *ACSS2* variant, NM_018677.4:c.1487 T > C p.(Val496Ala), plus a variant in the *PLEKHA7* gene, NM_001329630.2:c.617 T > C p.(Leu206Pro). Similarly, in proband C40 [Family 18], also a non-syndromic individual, the recurrent *ACSS2*(NM_018677.4):c.1487 T > C variant was identified in conjunction to a variant in *PLEKHA5*, NM_001256470.2:c.352 C > T p.(Pro118Ser). Both *PLEKHA7* and *PLEKHA5* were identified using the Clefting panel, however *PLEKHA7* only has moderate evidence for this gene-disease association while *PLEKHA5* does not have enough evidence and therefore not yet used for genome interpretation. In contrast, *ACSS2* is not present in any of the gene panels used but has been investigated in the context of nsOFC.

## Discussion

This study underscores the utility of exome sequencing in uncovering rare variants that contribute to both syndromic and non-syndromic OFCs in a Colombian population. We identified 15 candidate variants across 15 genes in 11 of 20 families (55%). The implicated genes are predominantly involved in embryonic development, cellular adhesion, transcription regulation, chromatin remodelling, and mitochondrial function, processes that are critical for proper tissue formation and differentiation, particularly during craniofacial development.

### Strong candidate genes for OFC

Several variants identified in our cohort support the candidacy of genes not currently included in curated clefting panels but have plausible biological relevance to orofacial clefting.

*FGF10* encodes a signalling molecule essential for coordinated epithelial-mesenchymal interactions during development of several organs, such as lungs, limbs and palate [39]. In vivo mice studies have shown that interaction between FGF10 and its receptor FGFR2 in the adjacent epithelium is critical for activation of Sonic hedgehog (Shh) expression, which subsequently promotes mesenchymal proliferation during palate development [38]. Beyond its developmental role, *FGF10* has also been implicated in gene-gene interactions in individuals with non-syndromic OFC. Using data from 806 Chinese nsOFC proband-parent trios, Li et al. [41] identified a significant interaction between rs2330542 in *FGF10* and rs1946295 in *TBX5*, associated with a 39% increase in relative risk when both variants co-occur. Likewise, the cooccurrence of rs7704166 in *FGF10* and rs7085073 in *FGFR2* was linked to an increased nsOFC risk. In an independent study, Menezes et al. [42] investigated variants in FGF pathway genes and found that individuals with bilateral cleft lip and palate and associated tooth agenesis showed increased risk associated with the rs1448037 variant in *FGF10*, which was not observed in controls. Although these variants may appear clinically insignificant when assessed individually, this evidence highlights the importance of gene-gene interactions in the aetiology of complex disorders such as nsOFC. Additionally, it underscores the potential for pathogenic non-coding variation to be overlooked using current pathogenicity classification frameworks.

The *MID1* variant, NM_000381.4:c.1354 G > A p.(Gly452Ser), was previously reported in a single patient with Opitz GBBB syndrome and also detected in the mildly symptomatic mother and affected sibling [35]. While the index patient reported by Pinson et al. did not present with OFC, phenotypic heterogeneity was observed across affected family members, and OFC is often an additional defect seen in patients with *MID1* variants [36]. This supports the possibility that *MID1*-related disruptions may contribute to clefting in a subset of individuals.

In our cohort, a missense variant in *FLNA*, NM_001110556.2:c.5069 C > T p.(Thr1690Met), was identified in a female proband and affected father. *FLNA* variants are associated with a phenotypic spectrum of X-linked syndromes, many of which include OFCs in their clinical presentation and are lethal or severely pathogenic in males [37, 43]. The proband’s phenotype aligns with this spectrum, suggesting that the *FLNA* variant may explain her condition and further supports its relevance to OFC pathogenesis. However, the absence of syndromic features in the father raises the possibility that this variant may be hypomorphic or that he may be mosaic. It is also possible that variable expressivity may be modulated the subjects’ polygenic backgrounds, as evidenced by Yu et al [44] in a large three-generation family with non-syndromic CL/P.

### Variants in emerging or novel candidate genes in OFC

This work further supports the involvement of *ZFHX3, ZFHX4, PLEKHA5* and *PLEKHA7* in the aetiology of OFCs. *ZFHX4* encodes a transcription factor highly expressed in the developing brain of several species [45]. In murine models, loss of Zfhx4 function has been associated with cleft palate, with its expression in the palatal shelves suggesting a crucial role in palatal development [46]. In a study with 756 child-parent trios of European (*n* = 415), Colombian (*n *= 275) and Taiwanese (*n* = 125) ancestry, whole genome sequencing was performed to determine the contributions of coding de novo mutations (DNMs) to an individual’s OFC risk [5]. The authors proposed both *ZFHX3* and *ZFHX4* as contributors to the aetiology of OFCs and note that *ZFHX4* was only one of two genes with multiple loss of function DNMs. Interestingly, of the two *ZFHX4* de novo mutations identified; one was observed in an individual of Colombian origin. Further functional studies support *ZFHX4* and *ZFHX3* as clefting genes that should be considered in genetic testing [40, 47–49].

*PLEKHA7* and *PLEKHA5* play a role in maintaining epithelial cell adhesion and polarity, essential functions for epithelial tissue formation. Both genes encode components of the p120-catenin (p120Ctn) complex, which interacts with key adhesion proteins, including E-cadherin and Nectin1, both previously implicated in OFC [4]. Cox and colleagues found that pathogenic variants in *PLEKHA7* and *PLEKHA5* segregate in individuals with nsOFC, reinforcing these genes’ role in craniofacial development. The authors of the study also note that while certain variants in *PLEKHA5* and *PLEKHA7* are likely pathogenic, others may act as susceptibility alleles within a polygenic model for nsOFC. In probands C36 and C40, the *PLEKHA* variants were found alongside the NM_018677.4:c.1487 T > C p.(Val496Ala) variant in *ACCS2* further supporting this oligogenic model. Together with existing evidence, our data support the role of both genes as candidates for nsOFC.

Exome sequencing studies in Honduran populations have identified *ACSS2* as a gene harbouring rare variants that segregate with nsOFC in a dominant manner with incomplete penetrance. One such variant, NM_018677.4:c.1487 T > C p.(Val496Ala), was found to co-segregate with the disease phenotype across multiple families, suggesting a potential role in nsOFC pathogenesis [21]. Although this variant is relatively frequent in the Admixed American ancestry group in gnomAD (MAF = 0.01387), with eight homozygous individuals reported, its significant enrichment in nsOFC patients and proposed incomplete penetrance supports its candidacy as a risk factor rather than a fully penetrant pathogenic variant. To further elucidate the functional impact of the NM_018677.4:c.1487 T > C variant, in vivo modelling could help clarify its biological impact and potential role in craniofacial development.

Additionally, patient C4 was found to carry two de novo variants with available evidence of pathogenicity—one in *TTN* and another in *FGF10*. The patient’s phenotype comprises not only cleft lip and palate, but also pulmonary hypertension and patent ductus arteriosus. While *FGF10* is more clearly associated with the cleft phenotype and *TTN* with cardiac abnormalities, emerging evidence has also suggested a potential link between *TTN* and OFC phenotypes [50], which could point to a possible interaction between both genes in the manifestation of OFCs.

### Functional and clinical relevance of identified variants

Our findings emphasise the complexity of OFC genetics, particularly in non-syndromic individuals where multiple variants of uncertain significance (VUS) were identified in genes with emerging relevance to craniofacial development. While some probands carried variants in well-established OFC genes, others harboured combinations of variants in genes not currently included in curated panels. These observations suggest that both syndromic and non-syndromic OFCs may arise from a broader spectrum of genetic mechanisms than previously recognised.

Moreover, the recurrence of variants such as *ACCS2*(NM_018677.4):c.1487 T > C in multiple probands, despite its relatively high frequency in certain populations, raises questions about penetrance and population-specific risk. These findings emphasise the need for functional validation and improved annotation of variants in diverse populations as well as the limitations of current variant prioritisation frameworks, which may overlook pathogenic contributions from gene-gene interactions.

### Limitations and recommendations

The identification of these novel variants is a clear indication that the Colombian population and Latin American population in general, have not been as extensively studied as European/North American populations, and are underrepresented in population databases. We aimed to address this gap in knowledge and provide new insights into the genetic architecture of OFC; however, several limitations should be considered. Due to the nature of the cohort, ability to obtain trios was not always possible and hence identification of de novo variants or phasing of variants was limited. Additionally, further validation such as Sanger sequencing was not possible due to time and budget constraints. As with any study using ES, our ability to detect non-coding and structural variants was limited. This is reflected by the absence of significant findings in probands from 7 families (2, 3, 5, 9, 11, 19 and 20). The phenotypes of these probands may be related to structural, noncoding variants, or epigenetic and/or environmental factors unaccounted for in this study, particularly in the nsOFC individuals. Alternatively, they may result from the combined effect of multiple common variants with low individual impact, consistent with a polygenic background. Probands from families 2, 3, 5, and 9, had syndromic phenotypes, suggesting a need for more thorough investigations.

Given the complexity of OFCs, we believe employing a multi-omics approach is essential to understanding its aetiology. Integrating genetic data with epigenetic and transcriptomic analyses may uncover regulatory mechanism and modifier effects that contribute to phenotypic variability. It is essential to investigate non-coding regions, as demonstrated by Li et al. [41], who identified significant interactions in genes pertinent to OFC within the upstream regions of *FGF* genes. Together these multi-omic approaches represent a critical future direction for advancing mechanistic understanding of OFC, strengthening diagnostic interpretation, and paving the way for future therapeutic advances.

## Supplementary information

Supplemental Material

Supplemental Tables S5
