# Supplementary material for: Rare variants in embryonic development and cell signalling genes in syndromic and non-syndromic orofacial clefts: evidence from a Colombian Caribbean cohort
Source: J Hum Genet. 2026 Mar 30;71(7):443–51. doi: 10.1038/s10038-026-01466-x (PMC13303074; doi:10.1038/s10038-026-01466-x)
Supplement: Supplementary file 1 — Supplemental Material [file 10038_2026_1466_MOESM1_ESM.docx]

# Supplementary Figures


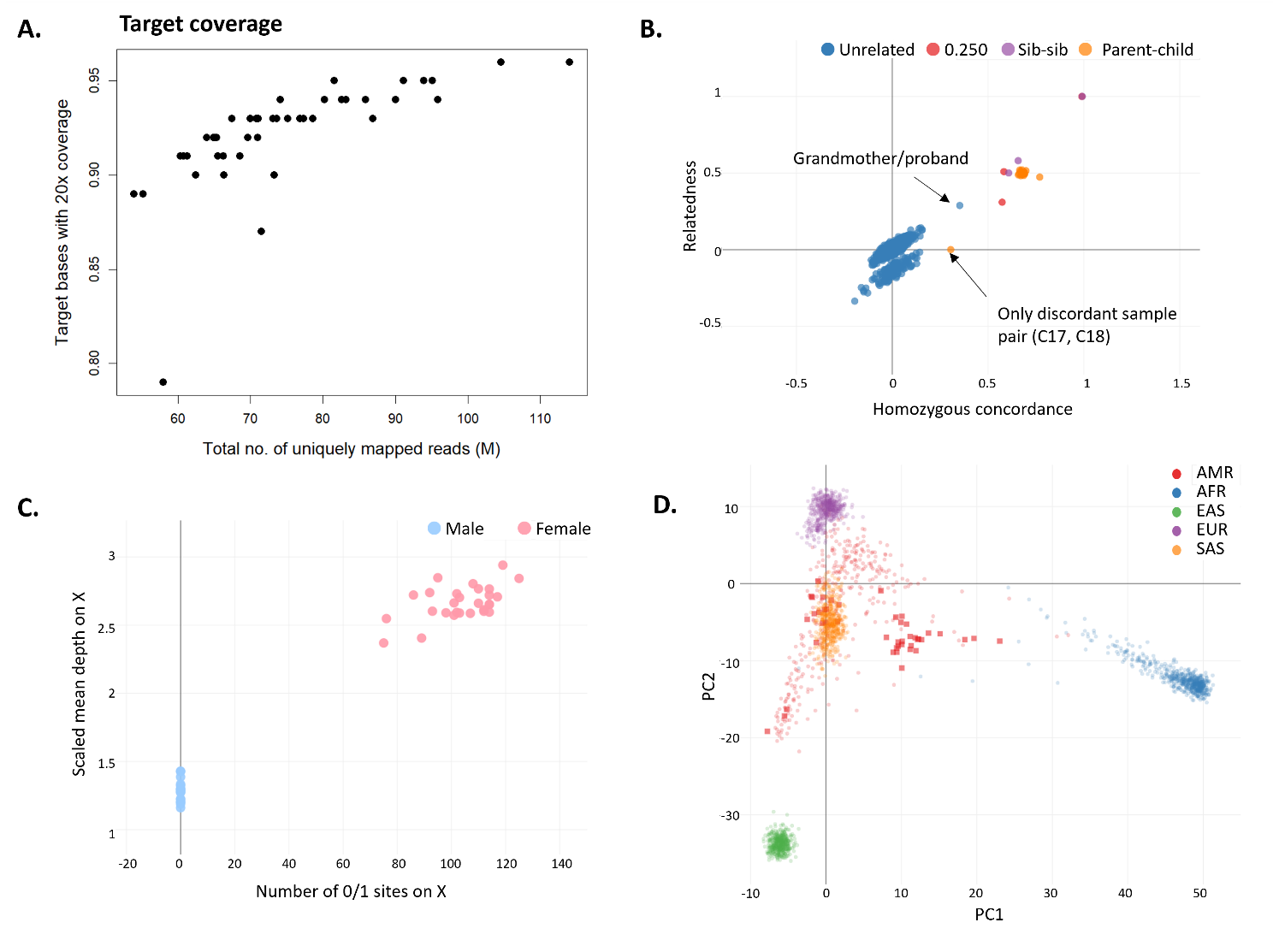


**Figure S1.** Quality assurance checks**. A.** Uniquely mapped reads vs percentage of target bases with 20x coverage, calculated using GATK’s DepthOfCoverage tool. **B.** Relatedness results by Somalier. All sample-sample pairs are plotted and coloured based on relatedness according to pedigree. Relatedness coefficient: 1=Identical twins/clones/duplicated; 0.5=parent/child/full sibling; 0.25 grandparent/grandchild; <0.20 distant relative/not related. Unrelated individuals are expected to have a homozygous concordance=0. **C.** Reported sex vs genotype inferred sex. Points are coloured based on reported sex. **D.** Principal Component Analysis (PCA) of ancestry estimation by Somalier. The PCA is generated using labelled samples from the 1000 Genomes project. Red squares represent our cohort (n=45) which cluster (labelled by Somalier) within the Admixed American Group (AMR: Red circles). African (AFR), East Asian (EAS), European (EUR), South Asian (SAS), and Admixed American (AMR).


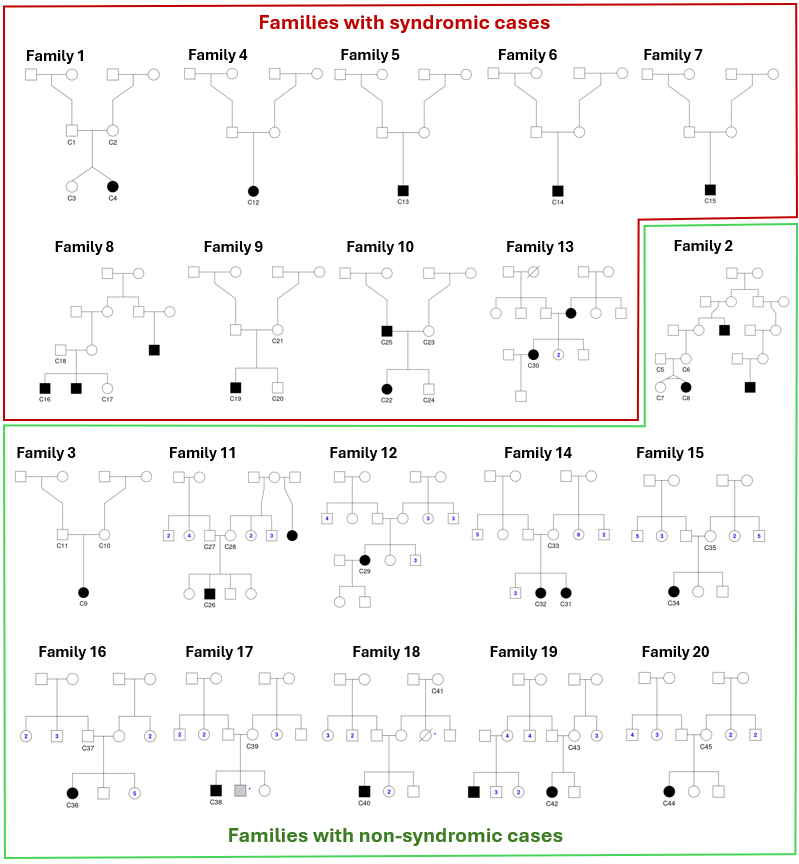


**Figure S2.** Pedigrees of recruited families. ID codes for research subjects appear under each square/circle, individuals whose DNA was collected and analysed. Symbols filled in black represent affected individuals. Numbers inside of circles and squares represent the number of brothers and sisters, respectively.

**Supplementary Tables**

# Supplemental Table S1. HPO terms for affected individuals.

| **Subject ID** | **Family** | **HPO Terms** |
| --- | --- | --- |
| C4 | 1 | Orofacial cleft (HP:0000202), Cleft palate (HP:0000175), Cleft lip (HP:0410030), Cleft upper lip (HP:0000204), Non-midline cleft lip (HP:0100335), Cleft maxillary alveolar ridge (HP:0010289), Unilateral cleft lip (HP:0100333), Cleft soft palate (HP:0000185), Unilateral alveolar cleft of maxilla (HP:0410033), Cleft hard palate (HP:0410005), Median cleft palate (HP:0009099), Unilateral cleft palate (HP:0100334), Gingival cleft (HP:0030690), Complete unilateral cleft lip (HP:5201001), Complete cleft maxillary alveolar ridge (HP:5201008), Complete cleft hard palate (HP:5201003), Pulmonary arterial hypertension (HP:0002092), Pulmonary venous hypertension (HP:0030950), Patent ductus arteriosus (HP:0001643) |
| C7 | 2 | Orofacial cleft (HP:0000202), Cleft palate (HP:0000175), Cleft lip (HP:0410030), Cleft upper lip (HP:0000204), Non-midline cleft lip (HP:0100335), Cleft maxillary alveolar ridge (HP:0010289), Unilateral cleft lip (HP:0100333), Cleft soft palate (HP:0000185), Unilateral alveolar cleft of maxilla (HP:0410033), Cleft hard palate (HP:0410005), Median cleft palate (HP:0009099), Unilateral cleft palate (HP:0100334), Gingival cleft (HP:0030690), Complete unilateral cleft lip (HP:5201001), Complete cleft maxillary alveolar ridge (HP:5201008), Complete cleft hard palate (HP:5201003), Anemia (HP:0001903) |
| C9 | 3 | Orofacial cleft (HP:0000202), Cleft palate (HP:0000175), Cleft lip (HP:0410030), Cleft upper lip (HP:0000204), Non-midline cleft lip (HP:0100335), Cleft maxillary alveolar ridge (HP:0010289), Unilateral cleft lip (HP:0100333), Cleft soft palate (HP:0000185), Unilateral alveolar cleft of maxilla (HP:0410033), Cleft hard palate (HP:0410005), Median cleft palate (HP:0009099), Unilateral cleft palate (HP:0100334), Gingival cleft (HP:0030690), Complete unilateral cleft lip (HP:5201001), Complete cleft maxillary alveolar ridge (HP:5201008), Complete cleft hard palate (HP:5201003) |
| C12 | 4 | Abnormal palate morphology (HP:0000174), High palate (HP:0000218), Seizure (HP:0001250), Porencephaly (ORPHA:2940 ), Periventricular leukomalacia (HP:0006970), Neurodevelopmental delay (HP:0012758), Persistent head lag (HP:0032988), Generalized hypotonia (HP:0001290) |
| C13 | 5 | Orofacial cleft (HP:0000202), Cleft palate (HP:0000175), Cleft soft palate (HP:0000185), Cleft hard palate (HP:0410005), Median cleft palate (HP:0009099), Unilateral cleft palate (HP:0100334), Complete cleft hard palate (HP:5201003), Scoliosis (HP:0002650), Lower limb asymmetry (HP:0100559), Upper limb asymmetry (HP:0100560), Abnormal facial shape (HP:0001999) |
| C14 | 6 | Orofacial cleft (HP:0000202), Cleft palate (HP:0000175), Cleft lip (HP:0410030), Cleft upper lip (HP:0000204), Non-midline cleft lip (HP:0100335), Cleft maxillary alveolar ridge (HP:0010289), Unilateral cleft lip (HP:0100333), Cleft soft palate (HP:0000185), Unilateral alveolar cleft of maxilla (HP:0410033), Cleft hard palate (HP:0410005), Median cleft palate (HP:0009099), Unilateral cleft palate (HP:0100334), Gingival cleft (HP:0030690), Complete unilateral cleft lip (HP:5201001), Complete cleft maxillary alveolar ridge (HP:5201008), Complete cleft hard palate (HP:5201003), Seizure (HP:0001250), Neurodevelopmental delay (HP:0012758), Hydrocephalus (HP:0000238), Agenesis of corpus callosum (HP:0001274), Decreased body weight (HP:0004325) |
| C15 | 7 | Orofacial cleft (HP:0000202), Cleft palate (HP:0000175), Cleft lip (HP:0410030), Cleft upper lip (HP:0000204), Non-midline cleft lip (HP:0100335), Cleft maxillary alveolar ridge (HP:0010289), Unilateral cleft lip (HP:0100333), Cleft soft palate (HP:0000185), Unilateral alveolar cleft of maxilla (HP:0410033), Cleft hard palate (HP:0410005), Median cleft palate (HP:0009099), Unilateral cleft palate (HP:0100334), Gingival cleft (HP:0030690), Complete unilateral cleft lip (HP:5201001), Complete cleft maxillary alveolar ridge (HP:5201008), Complete cleft hard palate (HP:5201003), Abnormal central motor function (HP:0011442), Kyphosis (HP:0002808), Tooth malposition (HP:0000692), Premature birth (HP:0001622), Pneumonia (HP:0002090), Global developmental delay (HP:0001263), Speech apraxia (HP:0011098), Intellectual disability (HP:0001249), Decreased body weight (HP:0004325) |
| C16 | 8 | Orofacial cleft (HP:0000202), Cleft palate (HP:0000175), Cleft lip (HP:0410030), Cleft upper lip (HP:0000204), Non-midline cleft lip (HP:0100335), Cleft maxillary alveolar ridge (HP:0010289), Cleft soft palate (HP:0000185), Cleft hard palate (HP:0410005), Median cleft palate (HP:0009099), Gingival cleft (HP:0030690), Complete cleft maxillary alveolar ridge (HP:5201008), Complete cleft hard palate (HP:5201003), Bilateral alveolar cleft of maxilla (HP:0410034), Bilateral cleft lip and palate (HP:0002744), Bilateral cleft lip (HP:0100336), Bilateral cleft palate (HP:0100337), Delayed speech and language development (HP:0000750), Abnormal oral frenulum morphology (HP:0000190), Small for gestational age (HP:0001518), Speech articulation difficulties (HP:0009088), Abnormal oral frenulum morphology (HP:0000190), Posteriorly rotated ears (HP:0000358) |
| C19 | 9 | Orofacial cleft (HP:0000202), Cleft lip (HP:0410030), Cleft upper lip (HP:0000204), Non-midline cleft lip (HP:0100335), Unilateral cleft lip (HP:0100333), Complete unilateral cleft lip (HP:5201001), Inguinal hernia (HP:0000023), Systolic heart murmur (HP:0031664), Intrauterine growth retardation (HP:0001511), Premature birth (HP:0001622), Small for gestational age (HP:0001518), Jaundice (HP:0000952), Short stature (HP:0004322) |
| C22 | 10 | Orofacial cleft (HP:0000202), Cleft palate (HP:0000175), Cleft lip (HP:0410030), Cleft upper lip (HP:0000204), Non-midline cleft lip (HP:0100335), Cleft maxillary alveolar ridge (HP:0010289), Unilateral cleft lip (HP:0100333), Cleft soft palate (HP:0000185), Unilateral alveolar cleft of maxilla (HP:0410033), Cleft hard palate (HP:0410005), Median cleft palate (HP:0009099), Unilateral cleft palate (HP:0100334), Gingival cleft (HP:0030690), Complete unilateral cleft lip (HP:5201001), Complete cleft maxillary alveolar ridge (HP:5201008), Complete cleft hard palate (HP:5201003), Premature birth (HP:0001622), Patent foramen ovale (HP:0001655), Astigmatism (HP:0000483), Delayed speech and language development (HP:0000750) |
| C25 | 10 | Orofacial cleft (HP:0000202), Cleft palate (HP:0000175), Cleft lip (HP:0410030), Cleft upper lip (HP:0000204), Non-midline cleft lip (HP:0100335), Cleft maxillary alveolar ridge (HP:0010289), Unilateral cleft lip (HP:0100333), Cleft soft palate (HP:0000185), Unilateral alveolar cleft of maxilla (HP:0410033), Cleft hard palate (HP:0410005), Median cleft palate (HP:0009099), Unilateral cleft palate (HP:0100334), Gingival cleft (HP:0030690), Complete unilateral cleft lip (HP:5201001), Complete cleft maxillary alveolar ridge (HP:5201008), Complete cleft hard palate (HP:5201003), Volvulus (HP:0002580) |
| C26 | 11 | Orofacial cleft (HP:0000202), Cleft palate (HP:0000175), Cleft lip (HP:0410030), Cleft upper lip (HP:0000204), Non-midline cleft lip (HP:0100335), Cleft maxillary alveolar ridge (HP:0010289), Unilateral cleft lip (HP:0100333), Cleft soft palate (HP:0000185), Unilateral alveolar cleft of maxilla (HP:0410033), Cleft hard palate (HP:0410005), Median cleft palate (HP:0009099), Unilateral cleft palate (HP:0100334), Gingival cleft (HP:0030690), Complete unilateral cleft lip (HP:5201001), Complete cleft maxillary alveolar ridge (HP:5201008), Complete cleft hard palate (HP:5201003), Shawl scrotum (HP:0000049) |
| C29 | 12 | Orofacial cleft (HP:0000202), Cleft lip (HP:0410030), Cleft upper lip (HP:0000204), Non-midline cleft lip (HP:0100335), Unilateral cleft lip (HP:0100333), Complete unilateral cleft lip (HP:5201001) |
| C30 | 13 | Orofacial cleft (HP:0000202), Cleft palate (HP:0000175), Cleft lip (HP:0410030), Cleft upper lip (HP:0000204), Non-midline cleft lip (HP:0100335), Cleft maxillary alveolar ridge (HP:0010289), Unilateral cleft lip (HP:0100333), Cleft soft palate (HP:0000185), Unilateral alveolar cleft of maxilla (HP:0410033), Cleft hard palate (HP:0410005), Median cleft palate (HP:0009099), Unilateral cleft palate (HP:0100334), Gingival cleft (HP:0030690), Complete unilateral cleft lip (HP:5201001), Complete cleft maxillary alveolar ridge (HP:5201008), Complete cleft hard palate (HP:5201003), Clinodactyly of the 5th finger (HP:0004209), Dental crowding (HP:0000678) |
| C31 | 14 | Orofacial cleft (HP:0000202), Cleft palate (HP:0000175), Cleft lip (HP:0410030), Cleft upper lip (HP:0000204), Non-midline cleft lip (HP:0100335), Cleft maxillary alveolar ridge (HP:0010289), Unilateral cleft lip (HP:0100333), Cleft soft palate (HP:0000185), Unilateral alveolar cleft of maxilla (HP:0410033), Cleft hard palate (HP:0410005), Median cleft palate (HP:0009099), Unilateral cleft palate (HP:0100334), Gingival cleft (HP:0030690), Complete unilateral cleft lip (HP:5201001), Complete cleft maxillary alveolar ridge (HP:5201008), Complete cleft hard palate (HP:5201003) |
| C32 | 14 | Orofacial cleft (HP:0000202), Cleft lip (HP:0410030), Cleft upper lip (HP:0000204), Non-midline cleft lip (HP:0100335), Unilateral cleft lip (HP:0100333), Complete unilateral cleft lip (HP:5201001) |
| C34 | 15 | Orofacial cleft (HP:0000202), Cleft palate (HP:0000175), Cleft lip (HP:0410030), Cleft upper lip (HP:0000204), Non-midline cleft lip (HP:0100335), Cleft maxillary alveolar ridge (HP:0010289), Unilateral cleft lip (HP:0100333), Cleft soft palate (HP:0000185), Unilateral alveolar cleft of maxilla (HP:0410033), Cleft hard palate (HP:0410005), Median cleft palate (HP:0009099), Unilateral cleft palate (HP:0100334), Gingival cleft (HP:0030690), Complete unilateral cleft lip (HP:5201001), Complete cleft maxillary alveolar ridge (HP:5201008), Complete cleft hard palate (HP:5201003), Recurrent otitis media (HP:0000403) |
| C36 | 16 | Orofacial cleft (HP:0000202), Cleft palate (HP:0000175), Cleft lip (HP:0410030), Cleft upper lip (HP:0000204), Non-midline cleft lip (HP:0100335), Cleft maxillary alveolar ridge (HP:0010289), Cleft soft palate (HP:0000185), Cleft hard palate (HP:0410005), Median cleft palate (HP:0009099), Gingival cleft (HP:0030690), Complete cleft maxillary alveolar ridge (HP:5201008), Complete cleft hard palate (HP:5201003), Bilateral alveolar cleft of maxilla (HP:0410034), Bilateral cleft lip and palate (HP:0002744), Bilateral cleft lip (HP:0100336), Bilateral cleft palate (HP:0100337) |
| C38 | 17 | Orofacial cleft (HP:0000202), Cleft palate (HP:0000175), Cleft lip (HP:0410030), Cleft upper lip (HP:0000204), Non-midline cleft lip (HP:0100335), Cleft maxillary alveolar ridge (HP:0010289), Unilateral cleft lip (HP:0100333), Cleft soft palate (HP:0000185), Unilateral alveolar cleft of maxilla (HP:0410033), Cleft hard palate (HP:0410005), Median cleft palate (HP:0009099), Unilateral cleft palate (HP:0100334), Gingival cleft (HP:0030690), Complete unilateral cleft lip (HP:5201001), Complete cleft maxillary alveolar ridge (HP:5201008), Complete cleft hard palate (HP:5201003) |
| C40 | 18 | Orofacial cleft (HP:0000202), Cleft palate (HP:0000175), Cleft lip (HP:0410030), Cleft upper lip (HP:0000204), Non-midline cleft lip (HP:0100335), Cleft maxillary alveolar ridge (HP:0010289), Unilateral cleft lip (HP:0100333), Cleft soft palate (HP:0000185), Unilateral alveolar cleft of maxilla (HP:0410033), Cleft hard palate (HP:0410005), Median cleft palate (HP:0009099), Unilateral cleft palate (HP:0100334), Gingival cleft (HP:0030690), Complete unilateral cleft lip (HP:5201001), Complete cleft maxillary alveolar ridge (HP:5201008), Complete cleft hard palate (HP:5201003) |
| C42 | 19 | Orofacial cleft (HP:0000202), Cleft palate (HP:0000175), Cleft lip (HP:0410030), Cleft upper lip (HP:0000204), Non-midline cleft lip (HP:0100335), Cleft maxillary alveolar ridge (HP:0010289), Unilateral cleft lip (HP:0100333), Cleft soft palate (HP:0000185), Unilateral alveolar cleft of maxilla (HP:0410033), Cleft hard palate (HP:0410005), Median cleft palate (HP:0009099), Unilateral cleft palate (HP:0100334), Gingival cleft (HP:0030690), Complete unilateral cleft lip (HP:5201001), Complete cleft maxillary alveolar ridge (HP:5201008), Complete cleft hard palate (HP:5201003), |
| C44 | 20 | Orofacial cleft (HP:0000202), Cleft palate (HP:0000175), Cleft lip (HP:0410030), Cleft upper lip (HP:0000204), Non-midline cleft lip (HP:0100335), Cleft maxillary alveolar ridge (HP:0010289), Cleft soft palate (HP:0000185), Cleft hard palate (HP:0410005), Median cleft palate (HP:0009099), Gingival cleft (HP:0030690), Complete cleft maxillary alveolar ridge (HP:5201008), Complete cleft hard palate (HP:5201003), Bilateral alveolar cleft of maxilla (HP:0410034), Bilateral cleft lip and palate (HP:0002744), Bilateral cleft lip (HP:0100336), Bilateral cleft palate (HP:0100337) |

# Supplemental Table S2. Clinical information of each individual recruited in this study. The last two columns show other affected systems. 1. CNS&MA: Central Nervous System and Mental Abilities, 2. CV: Cardiovascular, 3. MS: Musculoskeletal, 4. RS: Respiratory system, 5. “Other” include: preterm birth, inguinal hernia, intrauterine growth retardation, jaundice, astigmatism and volvulus. A complete list of phenotypic terms (with HPO codes) per proband is included in Supplemental Table S1. OFC: orofacial cleft, sOFC: syndromic OFC, nsOFC: nonsyndromic OFC, CPO: cleft palate only.

| **Family ID** | **Subject ID** | **Sex** | **Kinship** | **Family history** | **Type** | **Cleft description** | **CNS&MA^1^** | **CV^2^** | **MS^3^** | **RS^4^** | **Other^5^** |
| --- | --- | --- | --- | --- | --- | --- | --- | --- | --- | --- | --- |
| 1 | C1 | M | Father | No |  | Unaffected |  |  |  |  |  |
|  | C2 | F | Mother | No |  | Unaffected |  |  |  |  |  |
|  | C3 | F | Twin sister | No |  | Unaffected |  |  |  |  |  |
|  | C4 | F | Proband | No | sOFC | Right cleft lip and complete cleft palate |  | + |  |  |  |
| 2 | C5 | M | Father | Yes |  | Unaffected |  |  |  |  |  |
|  | C6 | F | Mother | Yes |  | Unaffected |  |  |  |  |  |
|  | C7 | F | Proband | Yes | nsOFC | Right cleft lip and complete cleft palate |  |  |  |  |  |
|  | C8 | F | Twin sister | Yes |  | Unaffected |  |  |  |  |  |
| 3 | C9 | F | Proband | No | nsOFC | Right cleft lip and complete cleft palate |  |  |  |  |  |
|  | C10 | F | Mother | No |  | Unaffected |  |  |  |  |  |
|  | C11 | M | Father | No |  | Unaffected |  |  |  |  |  |
| 4 | C12 | F | Proband | No | sOFC | Deep ogival palate (CPO) | + |  | + |  |  |
| 5 | C13 | M | Proband | No | sOFC | Cleft palate (CPO) |  |  | + |  |  |
| 6 | C14 | M | Proband | No | sOFC | Left cleft lip and complete cleft palate | + |  | + |  |  |
| 7 | C15 | M | Proband | No | sOFC | Left cleft lip and complete cleft palate | + |  | + | + | + |
| 8 | C16 | M | Proband | Yes | sOFC | Bilateral cleft lip and complete cleft palate | + |  | + |  |  |
|  | C17 | F | Sister | Yes |  | Unaffected |  |  |  |  |  |
|  | C18 | M | Father | Yes |  | Unaffected |  |  |  |  |  |
| 9 | C19 | M | Proband | No | sOFC | Left cleft lip |  | + | + |  | + |
|  | C20 | M | Twin brother | No |  | Unaffected |  |  |  |  |  |
|  | C21 | F | Mother | No |  | Unaffected |  |  |  |  |  |
| 10 | C22 | F | Proband | Yes | sOFC | Right cleft lip and complete cleft palate | + | + |  |  | + |
|  | C23 | F | Mother | Yes |  | Unaffected |  |  |  |  |  |
|  | C24 | M | Twin brother | Yes |  | Unaffected |  |  |  |  |  |
|  | C25 | M | Father | Yes | sOFC | Left cleft lip and complete cleft palate |  |  |  |  | + |
| 11 | C26 | M | Proband | Yes | nsOFC | Left cleft lip and complete cleft palate |  |  |  |  |  |
|  | C27 | M | Father | Yes |  | Unaffected |  |  |  |  |  |
|  | C28 | F | Mother | Yes |  | Unaffected |  |  |  |  |  |
| 12 | C29 | F | Proband | No | nsOFC | Left cleft lip |  |  |  |  |  |
| 13 | C30 | F | Proband | Yes | sOFC | Right cleft lip and complete cleft palate |  |  | + |  |  |
| 14 | C31 | F | Proband | No | nsOFC | Right cleft lip and complete cleft palate |  |  |  |  |  |
|  | C32 | F | Proband | No | nsOFC | Left cleft lip w/o alveolar cleft |  |  |  |  |  |
|  | C33 | F | Mother | No |  | Unaffected |  |  |  |  |  |
| 15 | C34 | F | Proband | No | nsOFC | Right cleft lip and complete cleft palate |  |  |  |  |  |
|  | C35 | F | Mother | No |  | Unaffected |  |  |  |  |  |
| 16 | C36 | F | Proband | No | nsOFC | Bilateral cleft lip and complete cleft palate |  |  |  |  |  |
|  | C37 | M | Father | No |  | Unaffected |  |  |  |  |  |
| 17 | C38 | M | Proband | No | nsOFC | Left cleft lip and complete cleft palate |  |  |  |  |  |
|  | C39 | F | Mother | No |  | Unaffected |  |  |  |  |  |
| 18 | C49 | M | Proband | No | nsOFC | Right cleft lip and complete cleft palate |  |  |  |  |  |
|  | C41 | F | Grandmother | No |  | Unaffected |  |  |  |  |  |
| 19 | C42 | F | Proband | Yes | nsOFC | Left cleft lip and complete cleft palate |  |  |  |  |  |
|  | C43 | F | Mother | No |  | Unaffected |  |  |  |  |  |
| 20 | C44 | F | Proband | No | nsOFC | Bilateral cleft lip and complete cleft palate |  |  |  |  |  |
|  | C45 | F | Mother | No |  | Unaffected |  |  |  |  |  |

# Supplemental Table S3. ACMG classification of candidate variants

| **Variant** | **Symbol** | **Existing variation** | **HGMC criteria** | **HGMD Class** |
| --- | --- | --- | --- | --- |
| NM_001267550.2:c.15796C>T p.(Arg5266*) | TTN | rs372277017  COSV100633043  ClinVar ID: 130662 | PVS1, PS2, PM2_supporting | P |
| NM_004465.2:c.355G>A p.(Gly119Arg) | FGF10 | rs1740186895  COSV99240410 | PS2, PM2_supporting, PP3 | LP |
| NM_001170535.3:c.968G>T p.(Ser323Ile) | ATAD3A | - | PM2_supporting, BP4 | VUS |
| NM_006734.4:c.5312A>G p.(Glu1771Gly) | HIVEP2 | rs763685280 | PM2_supporting, BP1 | VUS |
| NM_182641.4:c.658G>A p.(Glu220Lys) | BPTF | - | PM2_supporting, PP3, BP1 | VUS |
| NM_000381.4:c.1354G>A p.(Gly452Ser) | MID1 | rs1556004404  ClinVar ID: 546092 | PM2_supporting, PP1, BP1 | VUS |
| NM_001110556.2:c.5069C>T p.(Thr1690Met) | FLNA | rs782352193  COSV61036621  ClinVar ID: 591330 | PM2_supporting | VUS |
| NM_002633.3:c.734C>T p.(Ala245Val) | PGM1 | rs929270819  ClinVar ID: 1302539 | PM2_supporting | VUS |
| NM_001287491.2:c.946C>T p.(Pro316Ser) | TET3 | - | PM2_supporting, BP4 | VUS |
| NM_024721.5:c.6677A>C p.(Lys2226Thr) | ZFHX4 | - | PM2_supporting, PP3, BP1 | VUS |
| NM_018677.4:c.1487T>C p.(Val496Ala) | ACSS2 | rs59088485  ClinVar ID: 617833 | PS4, PP1, PP3, BS2 | VUS |
| NM_000138.5:c.1125G>T p.(Glu375Asp) | FBN1 | rs2043845159 | PM2_supporting, PP2 | VUS |
| NM_138927.4:c.78-4_78del p.? | SON | - | PVS1, PM2_supporting | LP |
| NM_001329630.2:c.617T>C p.(Leu206Pro) | PLEKHA7 | rs776711368 | PM2_supporting, PP3 | VUS |
| NM_001256470.2:c.352C>T p.(Pro118Ser) | PLEKHA5 | rs911407536 | PM2_supporting, BP4 | VUS |

# Supplemental Table S4. Clinical information available for variants filtered in our cohort

| **Sample ID / Family ID** | **Type of OFC** | **Variant** | **Variant-specific publications** | **ClinVar Reports** |
| --- | --- | --- | --- | --- |
| C4 / 1 | sOFC | NM_001267550.2(TTN):c.15796C>T p.(Arg5266*) | PMID 25589632: mentioned in the supplemental material Table S8. TTN truncating variants in publicly available control populations | RCV000118735.6:Genetic Services Laboratory, University of Chicago: Tibial muscular dystrophy, tardive, Pathogenic  RCV000807735.8: Labcorp Genetics: Dilated cardiomyopathy 1G- Autosomal recessive limb-girdle muscular dystrophy type 2J, VUS |
| C16 / 8 | sOFC | NM_000381.4(MID1):c.1354G>A p.(Gly452Ser) | PMID:18360914 (Opitz Syndrome), PMID:15121778 (Opitz syndrome) | RCV000657884.3: GeneDx, does not provide any condition: Pathogenic |
| C22 / 10 | nsOFC | NM_001110556.2(FLNA):c.5069C>T p.(Thr1690Met) | No | RCV000722508.2: New York Genome Center, Generalized-onset seizure, VUS  Gharavi Laboratory, Columbia University, no condition provided, VUS  RCV001862119.6: Labcorp Genetics, Oto-palato-digital syndrome, type II, Heterotopia, periventricular, X-linked dominant, Frontometaphyseal dysplasia  Melnick-Needles syndrome, Benign |
| C29 / 12 | nsOFC | NM_002633.3(PGM1):c.734C>T p.(Ala245Val) | No | RCV002032757.5: Labcorp Genetics, PGM1-congenital disorder of glycosylation, VUS  RCV001754428.5: Mayo Clinic Laboratories, No condition provided, VUS |
| C31 / 14 | nsOFC | NM_018677.4(ACSS2):c.1487T>C p.(Val496Ala) | PMID:28543373 (Cleft lip and palate), PMID:27229527 (Nonsyndromic cleft lip and palate) | RCV000755123.1: Center for Mendelian Genomics, University of Washington, Nonsyndromic cleft lip palate, Pathogenic (Same variant present in 3 different families)  RCV004555874.1: Clinical Genomics Laboratory, Washington University in St. Louis, no condition provided, VUS |
